# Vestibular‐Visual Reweighting in Persistent Postural‐Perceptual Dizziness: A Multilevel Resting‐State fMRI Study

**DOI:** 10.1155/np/9968808

**Published:** 2026-04-08

**Authors:** Jin-Ju Kang, Yongseon Yoo, Sang-Yeon Kim, Sohui Kim, Jong-Min Lee, Sun-Young Oh

**Affiliations:** ^1^ Department of Neurology, Jeonbuk National University Hospital and School of Medicine, Jeonju, Republic of Korea; ^2^ Research Institute of Clinical Medicine of Jeonbuk National University-Biomedical Research Institute of Jeonbuk National University Hospital, Jeonju, Republic of Korea, cuh.co.kr; ^3^ Department of Electronic Engineering, Hanyang University, Seoul, Republic of Korea, hanyang.ac.kr; ^4^ Department of Artificial Intelligence, Hanyang University, Seoul, Republic of Korea, hanyang.ac.kr; ^5^ Department of Biomedical Engineering, Hanyang University, Seoul, Republic of Korea, hanyang.ac.kr

**Keywords:** ALFF/fALFF biomarker, brainstem–cerebellar dysconnectivity, functional network connectivity, multisensory integration, persistent postural-perceptual dizziness, resting-state functional MRI, visual-cerebellar coupling

## Abstract

Persistent postural‐perceptual dizziness (PPPD) is a disabling functional vestibular disorder characterized by chronic dizziness and visually and motion‐induced unsteadiness that markedly impairs daily activities, yet it lacks objective neurobiological markers. We acquired resting‐state functional MRI (rs‐fMRI) in 52 patients with PPPD and 50 age‐ and sex‐matched healthy controls (HCs) and analyzed the data using a three‐tier approach: (i) functional network connectivity (FNC) of independent component analysis (ICA‐FNC), (ii) voxel‐wise measures of spontaneous amplitude of low‐frequency fluctuations (ALFF) and fractional ALFF (fALFF), and (iii) seed‐based connectivity using a priori vestibular and subcortical regions of interest (ROIs; e.g., cerebellar (CB) nodulus, parafascicular thalamus, and caudate). Integrating these analytic tiers, we observed a coherent pattern: broadly increased connectivity of CB and primary visual (VIS) networks together with selective hypoconnectivity between a brainstem–cerebellar (BSC) component and the multimodal vestibular cortex (MVC), oculomotor (frontal eye field [FEF]), and default‐mode networks (DMN). Voxel metrics revealed decreased ALFF in parietal and frontal opercular cortices—key vestibular integration regions—contrasting with increased fALFF in mid‐cingulate, lateral occipital, and premotor areas. Seed‐based mapping identified strengthened thalamo‐VIS, striato‐limbic, and nodulus‐hippocampal connectivity. Importantly, increased BSC‐to‐VIS coupling correlated positively with depressive symptom severity and state anxiety, but negatively with balance confidence and psychological resilience, linking network imbalance to the biopsychosocial phenotype of PPPD. These findings support a multiscale signature of vestibular cortical disengagement accompanied by maladaptive VIS‐CB reinforcement and motivate multicenter validation of network‐level markers as adjuncts to symptom‐based diagnosis.

## 1. Introduction

Persistent postural‐perceptual dizziness (PPPD) is a chronic functional vestibular disorder characterized by persistent dizziness, nonspinning vertigo, and visually induced postural unsteadiness that markedly impairs daily activities lasting 3 months or longer (diagnostic criteria in Table 1) [1, 2]. The condition was formally operationalized as a diagnostic entity by the Bárány Society in 2017, representing a consolidation of previously overlapping syndromes, including chronic subjective dizziness, phobic postural vertigo, and visual (VIS) vertigo [1, 3]. Core diagnostic features include symptoms that are exacerbated by upright posture, active or passive motion, and exposure to complex or moving VIS stimuli [1]. PPPD frequently develops following acute vestibular insults, anxiety disorders, or other precipitating events, and is commonly associated with psychiatric comorbidities, including anxiety and depression [2, 4]. Despite marked disability, conventional neuro‐otologic testing and structural MRI are usually normal or only minimally abnormal [5, 6].

**Table 1:** Bárány society diagnostic criteria for persistent postural‐perceptual dizziness (PPPD).

A. One or more symptoms of dizziness, unsteadiness, or nonspinning vertigo are present on most days for 3 months or more. 1. Symptoms last for prolonged (hours long) periods of time, but may wax and wane in severity. 2. Symptoms need not be present continuously throughout the entire day.
B. Persistent symptoms occur without specific provocation, but are exacerbated by 3 factors. 1. Upright posture. 2. Active or passive motion without regard to direction or position. 3. Exposure to moving visual stimuli or complex visual patterns.
C. The disorder is precipitated by conditions that cause vertigo, unsteadiness, dizziness, or problems with balance including acute, episodic, or chronic vestibular syndromes, other neurologic or medical illnesses, or psychological distress. 1. When the precipitant is an acute or episodic condition, symptoms settle into the pattern of criterion A as the precipitant resolves, but they may occur intermittently at first, and then consolidate into a persistent course. 2. When the precipitant is a chronic syndrome, symptoms may develop slowly at first and worsen gradually.
D. Symptoms cause significant distress or functional impairment.
E. Symptoms are not better accounted for by another disease or disorder.

*Note:* Reprinted from Staab et al. [1]. *J Vestib Res* 27 (4):191–208

Current models emphasize maladaptive sensory reweighting and postural control after an initial precipitating event (e.g., acute vestibular disorders, medical illness, or psychological distress) [1, 4]. Patients may develop increased VIS dependence, heightened body vigilance, and threat‐related appraisal, which together sustain a mismatch between perceived and objective stability [6–9]. Within this framework, cerebellar (CB) and brainstem–cerebellar (BSC) circuits are plausible substrates for persistent reweighting because they contribute to vestibular adaptation and predictive calibration of sensory consequences; CB involvement has been noted in several PPPD Resting‐state functional MRI (rs‐fMRI) studies [10–14]. Anxiety‐related traits (including neuroticism) and comorbid anxiety/depression are repeatedly associated with symptom persistence and handicap, suggesting that affective factors interact with altered sensory processing rather than serving as epiphenomena [9, 15, 16].

rs‐fMRI studies increasingly implicate disrupted visuo‐vestibular and limbic networks in PPPD [12, 17]. Prior reports commonly describe reduced activity or connectivity within multimodal vestibular cortical hubs (e.g., parietal/frontal opercula and insula), alongside increased engagement of VIS and prefrontal systems involved in attention and emotional regulation [10, 12, 17, 18]. Network‐level approaches further suggest abnormal interactions among CB, default‐mode network (DMN), and sensorimotor networks, consistent with compensatory sensory reweighting and postural hypervigilance [18–20]. However, findings remain heterogeneous across cohorts and analytic pipelines, and few studies have examined whether network‐level dysconnectivity, regional oscillatory abnormalities, and circuit‐specific coupling converge within the same sample and relate to clinical symptom burden and self‐reported functional outcomes (e.g., dizziness handicap, balance confidence, and affective symptom measures) [18–20].

To address this gap, we applied a three‐tier rs‐fMRI framework in 52 patients with PPPD and 50 matched healthy controls (HC), combining independent component analysis (ICA)‐based functional network connectivity (FNC), voxel‐wise amplitude metrics (amplitude of low‐frequency fluctuations (ALFF) and fractional ALFF [fALFF]), and hypothesis‐driven seed‐based connectivity within vestibular–limbic circuits [21–27]. By integrating complementary analyses at network, regional, and circuit scales, we aimed to determine whether the rs‐fMRI signatures of PPPD converge across levels and relate these neurofunctional features to clinical symptom burden and self‐reported functional outcomes [12, 17]. We hypothesized that PPPD would show reduced coupling between BSC vestibular processing and cortical multisensory/oculomotor networks, accompanied by altered coupling involving CB and VIS systems, and that the magnitude of these alterations would covary with dizziness‐related handicap, balance confidence, and affective burden.

## 2. Methods

### 2.1. Participants

Between January 2020 and August 2022, we prospectively recruited 56 patients diagnosed with PPPD from the Dizziness Clinic at Jeonbuk National University Hospital, South Korea. All diagnoses were made by a senior neurotologist (Sun‐Young Oh) according to the Bárány Society diagnostic criteria (Table 1) [1]. Inclusion criteria were: (1) age 20–75 years, (2) symptom duration ≥3 months, and (3) eligibility for MRI scanning. A HC group of 50 individuals was recruited via institutional advertisements and frequency‐matched at the group level for age and sex. After screening and imaging quality control, the final analyzed sample comprised 52 PPPD patients (25 men/27 women; median age 56 years) and 50 HCs (22 men/28 women; median age 61 years). All participants were right‐handed. Global cognitive function was screened using the Korean Mini Mental State Examination, 2nd Edition (K‑MMSE‑2). Complete descriptive statistics and between‐group comparisons (including age, sex, handedness, education, and K‑MMSE‑2 scores) are provided in Table 2.

**Table 2:** Demographic, vestibular, and psychometric characteristics of the study population.

	PPPD (*n* = 52)	HC (*n* = 50)	*p*‐Value
Demographics			
Sex, male, *n* (%)	25 (48.1)	22 (44.0)	0.68
Age, years, median (95% CI)	56 (51–64)	61 (38–64)	0.571
Symptom duration, months, median (95% CI)	13 (8–25)	—	—
Education, years, median (95% CI)	12 (9–16)	12 (6–16)	0.663
MMSE (30 points), median (95%CI)	28.5 (27–30)	29 (27–30)	0.513
Right handedness, *n* (%)	52 (100%)	50 (100%)	—
Unsteadiness measurements			
Dizziness handicap inventory	27 (20–40)	1 (0–4)	<0.001
Functional (nine questions, 36 points)	14 (8–16)	0 (0–2)	<0.001
Emotional (nine questions, 36 points)	8 (6–10)	0 (0–0)	<0.001
Physical (seven questions, 28 points)	6 (6–10)	0 (0–2)	<0.001
Activities‐specific balance confidence scale (16 items, 100 points)	79.69 (66.87–86.25)	96.88 (93.13‐99.38)	<0.001
Fullerton advanced balance scale (10 items, 40 points)	34.5 (31–36)	39 (38–40)	<0.001
Psychiatry and personality assessments			
Patient Health Questionnaire‐9 (nine items, 27 points)	8 (6–11)	1 (0–2)	<0.001
State‐trait anxiety inventory × 1 (20 items, 80 points)	50 (42–54)	32 (29–34)	<0.001
Big five inventory			
Neuroticism (tendency toward pessimistic worry, eight items, 40 points)	23 (20–25)	18 (16–20)	0.001
Extraversion (outgoing nature, eight items, 40 points)	24 (23–26)	24.5 (23–28)	0.516
Openness (to new ideas and experiences, 10 items, 50 points)	32 (30–35)	29 (28–31)	0.056
Agreeableness (affability and warmth, nine items, 45 points)	32.5 (31–34)	35.5 (31–37)	0.189
Conscientiousness (diligence and dutifulness, nine items, 45 points)	32 (29–33)	33 (29–38)	0.077
General self‐efficacy (10 items, 40 points)	29 (28–30)	30 (28–31)	0.474
Brief resilience scale (six items, 30 points)	19 (17–21)	22 (21–23)	0.040

*Note:* Values are expressed as median (95% confidence interval). Statistical significance was calculated using Mann–Whitney U test.

Abbreviations: MMSE, mini‐mental state examination; PPPD, persistent postural perceptual dizziness.

Exclusion criteria for PPPD patients were: (1) current or historical neuro‐otological disorders likely to confound vestibular processing (e.g., Ménière’s disease and vestibular migraine); (2) major neurological or structural brain pathologies on anatomical MRI—including cerebral infarction, intracranial tumors, demyelinating lesions, or significant white matter hyperintensities (Fazekas scale ≥2)—and neurodegenerative disorders (e.g., Parkinson’s disease or Alzheimer’s disease) that could independently alter resting‐state functional activity; (3) history of major head trauma or sensorineural hearing loss >40 dB; (4) use of psychoactive medications, including antidepressants or benzodiazepines, within 1 month before enrollment. HC participants had no history of dizziness, headache, vestibular disorders, psychiatric illness, or current medication affecting neurological function. Normal vestibular function in controls was confirmed using caloric testing, video head impulse testing (vHIT), and vestibular evoked myogenic potentials (VEMPs).

All participants provided written informed consent prior to enrollment. The study protocol was approved by the Institutional Review Board of Jeonbuk National University Hospital (IRB Number 2020‐07‐004) and conducted in accordance with the Declaration of Helsinki.

### 2.2. Procedures and Clinical Assessments

This study utilized a prospective case–control design, and all participants underwent (i) standard neuro‐otological examination, (ii) high‐resolution structural MRI, and (iii) 6‐min resting‐state fMRI in the eyes‐closed condition. This condition was selected to minimize VIS interference and ocular artifacts, thereby facilitating the identification of intrinsic multisensory vestibular network dynamics, consistent with prior neuroimaging studies in chronic vestibular disorders [28–32]. Immediately after scanning, they completed a battery of validated questionnaires that captured affective burden (Patient Health Questionnaire‐9 [PHQ‐9], STAI‐X1), [33, 34] personality traits (big‐five inventory and brief resilience scale [BRS]) and balance‐related disability (dizziness handicap inventory (DHI) and activities‐specific balance confidence (ABC), and Situational Vertigo Questionnaire) [35–39]. Clinical assessors were blinded to imaging results, and imaging analysts were blinded to clinical scores.

The primary objectives were: (1) To characterize multilevel alterations in resting‐state brain function in PPPD, using a three‐tier analytic pipeline‐ICA‐FNC to capture large‐scale network interactions, voxel‐wise ALFF/fALFF to quantify regional oscillatory power, and seed‐based connectivity to probe circuit‐specific coupling. (2) To determine whether these functional imaging metrics covary with behavioral phenotypes, specifically affective load (depression and state anxiety), personality traits (neuroticism and resilience) and self‐perceived postural instability (balance confidence and visually induced vertigo).

### 2.3. Self‐Report Measures of Dizziness and Balance Function

We administered three validated instruments to quantify subjective dizziness severity, balance confidence, and functional mobility.

#### 2.3.1. *DHI*

The DHI is a 25‐item questionnaire assessing the impact of dizziness across three domains: physical (seven items), emotional (nine items), and functional (nine items). Each item is scored as 0 (no), 2 (sometimes), or 4 (yes), yielding a total score ranging from 0 to 100. Higher scores indicate greater perceived disability. Scores above 60 are typically associated with significant handicap and increased fall risk [35, 40].

#### 2.3.2. ABC

The ABC scale evaluates self‐perceived confidence in performing 16 common daily activities that require postural control (e.g., walking in crowds and reaching overhead). Confidence is rated on a scale from 0% (no confidence) to 100% (complete confidence). The average percentage score reflects overall balance confidence, with lower values indicating fear of falling or avoidance behaviors [39, 41].

#### 2.3.3. Fullerton Advanced Balance (FAB)

The FAB is a performance‐based measure consisting of 10 tasks designed to assess both static and dynamic balance in older adults and individuals with functional dizziness. Each task is scored on a five‐point scale (0–4), with a total maximum score of 40. Scores ≤25 indicate a higher risk of falling [42, 43].

To evaluate psychological factors associated with PPPD, we administered a battery of validated self‐report instruments addressing depression, anxiety, personality traits, resilience, and self‐efficacy.

#### 2.3.4. PHQ‐9

A nine‐item scale assessing depressive symptoms over the preceding 2 weeks. Each item is scored from 0 (not at all) to 3 (nearly every day), yielding a total score from 0 to 27. Higher scores reflect greater severity of depression. A score ≥10 suggests moderate depressive symptoms [33].

#### 2.3.5. State–Trait Anxiety Inventory X1 (STAI‐X1)

The STAI‐X1 measures transient (state) anxiety levels through 20 items, each rated on a four‐point Likert scale. Total scores range from 20 to 80, with higher values indicating greater anxiety. A score ≥40 is generally indicative of clinically relevant anxiety [34].

#### 2.3.6. Big Five Inventory

A 44‐item personality scale measuring five traits: neuroticism, extraversion, openness, agreeableness, and conscientiousness. Participants rate agreement on a five‐point Likert scale. Neuroticism, reflecting emotional instability and sensitivity to stress, was the primary trait of interest in this study [37].

#### 2.3.7. BRS

A six‐item scale designed to measure psychological resilience—the ability to recover from stress. Responses are rated on a five‐point Likert scale and averaged to produce a final score. Higher values indicate greater resilience [36, 44].

#### 2.3.8. General Self‐Efficacy (GSE)

This 10‐item scale assesses confidence in one’s ability to cope with difficult situations. Each item is rated from 1 (not at all true) to 4 (exactly true), yielding a total score ranging from 10 to 40. Higher scores indicate greater perceived self‐efficacy [38, 45].

### 2.4. Imaging Data Acquisition and Preprocessing

All participants underwent MRI scanning at Jeonbuk National University Hospital using a Philips Achieva 3.0 tesla MRI scanner. High‐resolution structural images were acquired using a 3D T1‐weighted turbo field echo (TFE) sequence (repetition time [TR] = 8.1 ms, echo time [TE] = 3.7 ms, flip angle = 8°, field of view = 240 mm, matrix = 256 × 256, 180 sagittal slices, slice thickness = 1.0 mm; isotropic voxel size = 1.0 × 1.0 × 1.0 mm^3^ [3]). Structural images were visually inspected by a neuroradiologist blinded to clinical diagnosis to screen for exclusionary findings (e.g., cerebral infarction, tumor, demyelinating lesions, or significant white matter hyperintensities; Fazekas scale ≥ 2).

rs‐fMRI was acquired immediately after the structural scan using a T2 ^∗^‐weighted echo‐planar imaging (EPI) sequence to measure blood–oxygen‐level‐dependent (BOLD) activity during eyes‐closed rest. Because PPPD symptoms are characteristically aggravated by VIS input and visually complex environments, we acquired rs‐fMRI with eyes closed to minimize external VIS stimulation and reduce oculomotor/VIS confounds, thereby facilitating the characterization of intrinsic vestibular and multisensory network dynamics. Acquisition parameters were: TR = 3000 ms, TE = 30 ms, flip angle = 90°, field of view = 220 mm, matrix = 64 × 64, 40 contiguous axial slices, slice thickness = 3 mm, native voxel size = 3.4 × 3.4 × 3.0 mm^3^ [3], and 130 volumes/scan (6 min 30 s). Participants were instructed to remain still, stay awake, and not engage in any structured mental task.

Preprocessing was performed using the Data Processing & Analysis for Brain Imaging (DPABI) toolbox45 (DPARSF module) based on SPM12 (Wellcome Trust Centre for Neuroimaging, London, UK; http://www.fil.ion.ucl.ac.uk/spm/) [46]. The first five volumes were discarded to allow for magnetization equilibrium. The remaining volumes underwent slice timing correction and realignment for head motion correction using a six‐parameter rigid‐body transformation [47]. Participants were excluded if head motion exceeded 2 mm translation or 2° rotation in any direction and/or if insufficient data remained after censoring. Field‐map‐based susceptibility distortion correction was not performed because field maps were not available. To reduce misregistration errors due to EPI geometric distortions, spatial normalization was performed using a standard EPI template [46, 48]. Functional images were normalized to Montreal Neurological Institute (MNI) space, resampled to 3 × 3 × 3 mm^3^ [3] isotropic voxels, and spatially smoothed using a 6 mm full‐width at half‐maximum (FWHM) Gaussian kernel.

To reduce physiological noise and residual motion effects, nuisance covariates were regressed from voxel‐wise time series following the motion‐control framework described by Jo et al. [49] Regressors included: (1) the six realignment parameters and their first‐order temporal derivatives (12 motion regressors) [47], (2) mean signals from white matter and cerebrospinal fluid masks, and (3) linear trends. Global signal regression was not performed. Residual motion outliers were addressed using scrubbing (censoring). Volumes with excessive motion, defined as framewise displacement (FD; Jenkinson) [50] >0.2 mm, were censored according to criteria described by Jo et al. and Power et al. [49, 51] For functional connectivity analyses (ICA‐FNC and seed‐based FC), the residual time series were temporally band‐pass filtered (0.009–0.08 Hz) [49]. ALFF/fALFF computations were performed without additional time‐domain band‐pass filtering, because fALFF requires the full frequency spectrum.

### 2.5. ICA

Group‐level ICA was performed using the Group ICA of fMRI Toolbox (GIFT; version 4.0 b) following the standard temporal‐concatenation framework [52]. Preprocessed functional images from all participants (PPPD and controls) were temporally concatenated to derive a common set of intrinsic connectivity networks (ICNs) that served as a shared spatial reference for cross‐subject comparisons; group‐level IC maps were not used for statistical inference. Subject‐specific spatial maps and time courses were obtained for each participant via GIFT back‐reconstruction and used for all subsequent between‐group analyses. Data were reduced using principal component analysis (PCA) and decomposed into [25] ICs using the Infomax algorithm [53]. Component stability was assessed using the Icasso toolbox (20 repetitions) [54].

IC selection was based on (1) VIS inspection, (2) spatial pattern matching with established resting‐state network templates, and (3) frequency‐domain inspection of component time courses. Components dominated by non‐gray‐matter signals (e.g., cerebrospinal fluid, white matter, and large vessels), prominent edge effects, or lacking a characteristic low‐frequency peak were classified as artifacts and excluded. While all 25 identified ICs were initially evaluated, we prioritized six ICNs for the final analysis based on their established roles in multisensory vestibular processing and the pathophysiology of PPPD. The rationale for selecting these specific nodes was as follows: (1) the DMN for its association with postural hypervigilance and self‐referential distress; (2) the VIS network to evaluate maladaptive sensory reweighting and VIS dependence; (3) the CB network for its role in fine‐tuning vestibular‐motor output and postural control; (4) the oculomotor/frontal eye field (FEF) network to investigate dysregulation in top–down control over ocular stability and gaze‐related anxiety, which are core features of visually induced dizziness in PPPD; (5) the multimodal vestibular cortex (MVC) network (posterior perisylvian regions, including OP2/PIC/TPJ) to assess the primary cortical hubs for integrating multisensory vestibular information; (6) a BSC network (ponto‐vermian) representing the subcortical relay and low‐level sensory gating station. Spatial maps for all 25 ICs are provided in Supporting Information 1: (Figure S1) [10, 12, 17, 18, 55–57].

### 2.6. FNC

FNC was computed from the subject‐specific (back‐reconstructed) IC time courses [26]. Time courses were despiked using the 3dDespike algorithm and low‐pass filtered (high‐frequency cutoff = 0.08 Hz; the 0.009 Hz high‐pass was imposed during preprocessing). Pearson correlation coefficients were calculated between all pairs of retained IC time courses (15 unique edges for 6 networks), yielding an individual symmetric FNC matrix for each participant. Correlation coefficients were Fisher *r*‐to‐*z* transformed for statistical analysis. Group differences in FNC were assessed in the Mancovan toolbox within GIFT using two‐sample *t*‐tests, with age, sex, and mean FD included as covariates of no interest; multiple comparisons were controlled using false discovery rate (FDR) correction at *q* < 0.05. Only connections surviving this correction were considered statistically significant.

### 2.7. Amplitude of Low Frequency Fluctuations (ALFF) and fALFF

Voxel‐wise ALFF and fALFF were computed using DPABI [46] to quantify spontaneous regional activity. After preprocessing and nuisance regression, each voxel’s time series was transformed into the frequency domain using fast Fourier transform (FFT). ALFF [22] was calculated as the averaged square root of the power spectrum within the low‐frequency band (0.01–0.08 Hz). fALFF [23] was computed as the ratio of power within the same low‐frequency band (0.01–0.08 Hz) to the total power across the full frequency range available for TR = 3 s (0–0.167 Hz; Nyquist frequency). Individual ALFF and fALFF maps were standardized into z‐scores (zALFF and zfALFF) by subtracting the whole‐brain mean and dividing by the standard deviation within the whole‐brain mask.

Voxel‐wise group comparisons between PPPD patients and HCs were performed in SPM12 using two‐sample *t*‐tests with age, sex, and mean FD as covariates [58]. Statistical significance was defined using cluster‐level family‐wise error (FWE) correction at p_FWE < 0.05, with a cluster‐forming threshold of *p*  < 0.001 (uncorrected).

### 2.8. Seed‐Based Functional Connectivity Analysis

To investigate circuit‐level alterations related to vestibular integration, affective regulation, and spatial memory in PPPD, seed‐based rs‐fMRI connectivity analyses were performed using a priori regions of interest (ROIs) defined in MNI space from validated neuroanatomical atlases and prior neurovestibular literature (Supporting Information 2: Table S2). The selection of specific seeds was informed by established neuroanatomical pathways and prior evidence of structural pathology in PPPD [55]. Specifically, the caudate nucleus was selected based on reported gray matter volume reductions in PPPD patients and its role in the transition from goal‐directed to automated balance control [59–61]. The parafascicular thalamic nucleus was included as a critical subcortical gatekeeper that relays vestibular information from the brainstem to the striatum, mediating non‐VIS spatial orientation [62, 63]. Cortical vestibular and association ROIs included bilateral OP2/parieto‐insular vestibular cortex (PIVC) masks derived from the Jülich histological atlas [55] and bilateral inferior parietal lobule (IPL) from the Harvard–Oxford Cortical Atlas [64]. CB ROIs (nodulus/lobule X and flocculus) were defined using the SUIT CB atlas [57]. Limbic and striatal ROIs (bilateral amygdala, hippocampus, parahippocampal gyrus, and caudate) were defined using the AAL3 atlas [65], whereas cortical affective/salience ROIs (anterior cingulate cortex [ACC], medial prefrontal cortex [mPFC; Harvard–Oxford “frontal medial cortex” parcel], and insula) were defined using the Harvard–Oxford atlas [64]. The parafascicular thalamic nucleus was defined using a stereotactic thalamic atlas (Morel/Krauth) [66]. For each seed ROI, the mean time series was extracted and correlated with the time series of every other voxel in the brain (Pearson correlation), yielding whole‐brain connectivity maps for each participant. Correlation coefficients were Fisher *r*‐to‐*z* transformed for group‐level inference. Group comparisons were conducted in SPM12 using independent two‐sample *t*‐tests, with age, sex, and mean FD included as covariates of no interest. Statistical significance was determined using cluster‐level FWE correction at pFWE < 0.05 with a cluster‐forming threshold of *p*  < 0.001 (uncorrected).

## 3. Statistical Analysis

### 3.1. Behavioral and Clinical Data Analysis

All behavioral and clinical data analyses were performed using IBM SPSS Statistics version 23.0 (IBM Corp., Armonk, NY, USA). Continuous variables are presented as medians with 95% confidence intervals (CIs), and categorical variables are presented as counts with percentages. Normality of continuous variables was assessed using the Shapiro–Wilk test. Between‐group differences in demographic and clinical characteristics were evaluated using Mann–Whitney U‐tests for nonnormally distributed continuous data and Pearson’s and chi^2^; tests for categorical data (or Fisher’s exact tests when expected cell counts were <5).

Within the PPPD group, associations between balance‐related measures (DHI, ABC, and FAB) and psychometric scores (PHQ‐9, STAI‐X1, BFI subscales, BRS, and GSE) were examined using Spearman’s rank correlation coefficients. All statistical tests were two‐tailed, and statistical significance was set at *p*  < 0.05.

### 3.2. Neuroimaging Data Analysis

All neuroimaging computations were performed in MATLAB R2023a (MathWorks, Natick, MA, USA) using Statistical Parametric Mapping (SPM12; Wellcome Trust Centre for Neuroimaging, London, UK), the DPABI toolbox (DPABI v6.2), the Group ICA of fMRI Toolbox (GIFT v4.0b), and custom scripts [52, 67–69].

### 3.3. ICA‐FNC

Group differences in FNC between PPPD patients and HCs were assessed using the Mancovan toolbox within GIFT [52]. Two‐sample *t*‐tests were conducted on Fisher *z*‐transformed FNC values (pairwise correlations between IC time courses) [26]. Age, sex, and mean FD were included as covariates of no interest [51]. To account for multiple comparisons across all network pairs (15 unique edges for six networks), FDR correction (Benjamini–Hochberg procedure) was applied at *q* < 0.05. Only connections surviving FDR correction were considered statistically significant [70].

### 3.4. Amplitude of ALFF and fALFF

Voxel‐wise group comparisons of standardized ALFF (zALFF) and fALFF (zfALFF) maps between PPPD patients and HCs were performed using two‐sample *t*‐tests implemented in SPM12 [71]. Age, sex, and mean FD were included as nuisance covariates in the general linear model [71]. Statistical significance was defined using cluster‐level FWE correction at pFWE < 0.05, with an initial cluster‐forming threshold of *p*  < 0.001 (uncorrected) [72].

### 3.5. Seed‐Based Functional Connectivity Analysis

Seed‐based functional connectivity analyses were conducted using SPM12 [71]. For each seed region, whole‐brain connectivity maps (Fisher *z*‐transformed correlation coefficients) were compared between PPPD patients and HCs using two‐sample *t*‐tests. Age, sex, and mean FD were included as covariates [51]. Statistical significance was determined using cluster‐level FWE correction at pFWE < 0.05, with a cluster‐forming threshold of *p*  < 0.001 (uncorrected) [72].

### 3.6. Brain‐Behavior Correlations

To examine the relationship between neuroimaging metrics and clinical phenotypes, significant imaging features (FNC edges, ALFF/fALFF clusters, and seed‐based connectivity measures) were correlated with clinical variables (e.g., DHI, ABC, PHQ‐9, STAI‐X1, BRS, and neuroticism) within the PPPD group. Partial Spearman rank correlations were computed, controlling for age, sex, and mean FD [51, 73]. To account for multiple comparisons across tested correlations, FDR correction (Benjamini–Hochberg procedure) was applied at *q* < 0.05. Only correlations surviving FDR correction were reported [70].

### 3.7. Quality Control and Motion Parameters

Head motion parameters were systematically evaluated and compared between groups using Mann–Whitney *U*‐tests. Mean FD (Jenkinson method) and maximum displacement were computed for all participants. Group differences in motion parameters were assessed to ensure that observed functional connectivity differences were not driven by differential head motion between PPPD patients and controls.

### 3.8. Missing Data

No imputation was applied for missing data, as missingness was less than 5% for all variables. Participants with incomplete data for specific analyses were excluded on a case‐by‐case basis.

## 4. Results

### 4.1. Demographic and Clinical Data

A total of 52 PPPD patients (median age: 56 years; 95% CI: 51–64) and 50 age‐ and sex‐matched HCs (median age: 61 years; 95% CI: 38–64) were analyzed (Supporting Information 2: Table S1). The two groups did not differ significantly in age, sex, or handedness.

PPPD patients primarily reported core vestibular symptoms described as sensations of wobbling (unsteadiness), feeling of falling, veering, floating, or drunkenness (Supporting Information 2: Table S1). Nonspinning dizziness was most common and was frequently accompanied by sensations of faintness or cloudiness, whereas spinning‐type dizziness was also reported in a subset of patients. Nineteen of 52 patients (36.5%) reported progressive worsening of symptoms overtime. Common precipitating factors included benign paroxysmal positional vertigo (BPPV), emotional stress, panic disorder, vestibular neuritis, Meniere’s disease, migraine, and other conditions such as syncope, anemia, and trauma. Symptom‐exacerbating conditions were systematically assessed through a structured interview and exhibited a characteristic profile: exacerbation occurred during upright posture (e.g., standing or sitting without back support), active or passive movement irrespective of direction (e.g., head rotation or vehicular travel), and exposure to visually complex environments (e.g., moving patterns, crowded spaces, digital screens, or fine print reading). Most patients reported symptom relief when adopting a lying or supported sitting posture, remaining stationary, or engaging in conversation.

Objective vestibular function tests showed generally normal results (Supporting Information 2: Table S1). Caloric testing revealed mild unilateral caloric paresis in several patients with a history of vestibular neuritis (ranging from −29.22% to 42.88%), but most other patients showed normal caloric responses. Similarly, vHIT for horizontal canal gain was within normal limits (≥0.70) for the majority, though isolated reduced gains were observed in specific cases. Ocular VEMPs and cervical VEMPs (oVEMPs/cVEMPs) were typically normal; delayed responses were identified in some individuals, predominantly unilaterally. Vestibular function was confirmed as normal in all controls through caloric testing (canal paresis < 25%), vHIT (gain ≥ 0.70 for all canals), and VEMPs (oVEMP and cVEMP within normal latency and amplitude ranges).

### 4.2. Self‐Perceived Unsteadiness and Psychological Profiles

**Figure 1:**
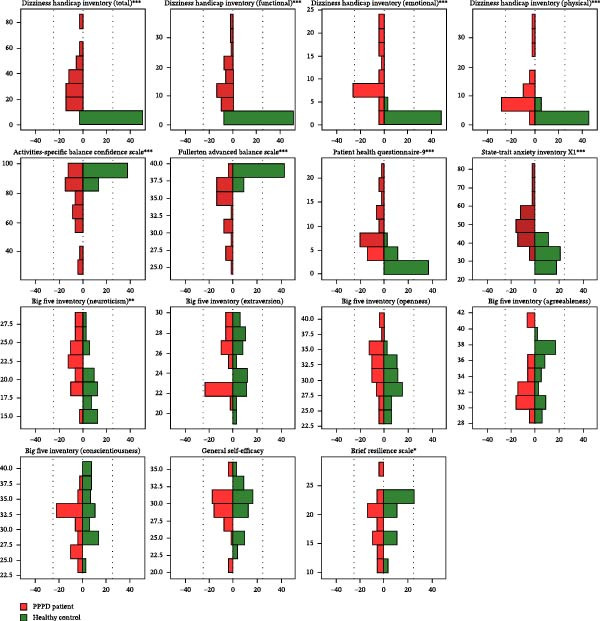
Mirror‐image histograms comparing self‐reported dizziness, balance, mood, personality, and coping measures between patients with persistent postural‐perceptual dizziness (PPPD) and healthy controls. Scores for each questionnaire are displayed as back‐to‐back frequency histograms, with PPPD patients shown on the left in red and controls on the right in green. The central vertical line represents zero on the abscissa (frequency = 0); bin width = 2 points for all instruments.  ^∗^ *p*  < 0.05,  ^∗∗^ *p*  < 0.01,  ^∗∗∗^ *p*  < 0.001.

Compared with HC, PPPD patients exhibited significantly greater perceived dizziness‐related disability (Table 2 and Figure 1). The DHI total scores were elevated in the PPPD group (median: 27; 95% CI: 20–40) relative to the control group (median: 1; 95% CI: 0–4), with significant differences across all subdomains: functional (14 vs. 0), emotional (8 vs. 0), and physical (6 vs. 0; all *p*  < 0.001). Consistent with these findings, the ABC scale indicated lower balance confidence among PPPD patients (median: 79.7%) compared with HC (median: 96.9%, *p*  < 0.001). Functional mobility, assessed via the FAB scale, was also reduced in the PPPD group (34.5 vs. 39, *p*  < 0.001).

Psychiatric evaluations revealed higher depressive symptoms (PHQ‐9 median: 8 vs. 1, *p*  < 0.001) and higher state anxiety (STAI‐X1 median: 50 vs. 32, *p*  < 0.001) among PPPD patients (Table 2 and Figure 1). Personality assessment using the BFI revealed that neuroticism was higher in the PPPD group (23 vs. 18; *p* = 0.001). No statistically significant group differences were observed in other personality domains (extraversion, openness, agreeableness, and conscientiousness). Psychological resilience, measured by the BRS, was lower in the PPPD group (19 vs. 22, *p* = 0.040). GSE scores did not differ significantly between the groups.

### 4.3. Correlation Between Psychological Traits and Unsteadiness

Within the PPPD cohort, Spearman correlation analyses demonstrated that higher PHQ‐9 scores were associated with higher DHI‐functional (*r* = 0.500, *p* = 0.009) and DHI‐emotional (*r* = 0.416, *p* = 0.035) subscores (Table 3). Higher BRS scores were associated with higher balance confidence (ABC: *r* = 0.572, *p* = 0.002) and lower DHI‐functional scores (*r* = −0.454, *p* = 0.020). Neuroticism was negatively correlated with ABC (*r* = −0.399, *p* = 0.043). Conscientiousness was positively correlated with DHI total (*r* = 0.483, *p* = 0.012) and DHI‐functional (*r* = 0.488, *p* = 0.011).

**Table 3:** Spearman correlations between balance measures and psychometric traits in PPPD.

	ABC		FAB		DHI							
					Total		Functional		Emotional		Physical	
	*r*	*p*	*r*	*p*	*r*	*p*	*r*	*p*	*r*	*p*	*r*	*p*
PHQ‐9	−0.306	0.128	−0.293	0.146	0.227	0.171	0.500	0.009	0.416	0.035	0.201	0.324
STAI‐X1	−0.316	0.116	−0.232	0.253	0.043	0.836	0.106	0.607	0.020	0.922	0.207	0.310
BFI‐neuroticism	−0.399	0.043	−0.248	0.222	−0.016	0.940	0.165	0.420	0.184	0.367	0.085	0.679
BFI‐extraversion	0.236	0.246	0.235	0.248	0.245	0.228	0.107	0.603	0.122	0.548	0.456	0.190
BFI‐openness	−0.156	0.445	0.104	0.615	0.238	0.162	0.090	0.664	0.011	0.957	−0.036	0.863
BFI‐agreeableness	0.037	0.858	−0.007	0.973	0.270	0.182	0.124	0.546	0.196	0.336	0.405	0.060
BFI‐conscientiousness	−0.088	0.671	0.289	0.153	0.483	0.012	0.488	0.011	0.167	0.414	0.037	0.856
GSE	0.313	0.119	0.157	0.443	0.159	0.437	0.074	0.719	−0.032	0.877	0.080	0.699
BRS	0.572	0.002	0.112	0.585	−0.253	0.212	−0.454	0.020	−0.352	0.078	−0.169	0.410

*Note:* The correlation between unsteadiness parameters and anxiety‐related personality was assessed using Spearman’s nonparametric bivariate correlation.

Abbreviations: ABC, activities‐specific balance confidence scale; BFI, big five inventory; BRS, brief resilience scale; DHI, dizziness handicap inventory; FAB, Fullerton advanced balance scale; GSE, general self‐efficacy; PHQ‐9, Patient Health Questionnaire‐9; STAI‐X1, state‐trait anxiety inventory X1.

### 4.4. Resting‐State Functional Connectivity Alterations

A total of 56 patients with PPPD and 50 HC were initially recruited. After predefined head‐motion quality control, 4 PPPD patients were excluded due to excessive motion (>2.0 mm translation or >2.0° rotation and/or mean FD >0.25 mm), resulting in a final cohort of 52 PPPD patients (median age: 56 years; 95% CI: 51–64) and 50 age‐ and sex‐matched HC (median age: 61 years; 95% CI: 38–64). Mean FD did not differ significantly between the final groups (*p*  = 0.44), and all included participants had mean FD <0.21 mm.

**Figure 2:**
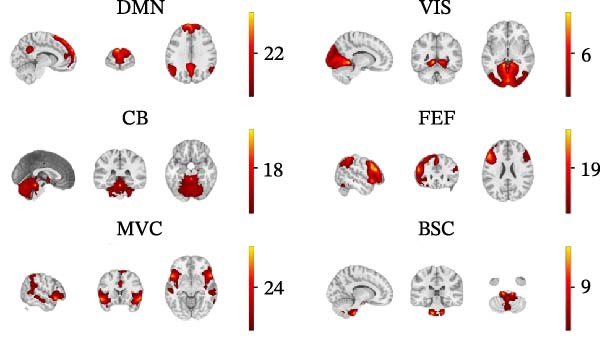
Spatial maps of the six intrinsic connectivity networks (ICNs) identified via group‐level independent component analysis (ICA). Across all participants, six networks relevant to multisensory and vestibular processing were selected and categorized as follows: the default mode network (DMN), visual network (VIS), cerebellar network (CB), oculomotor/frontal eye field network (FEF), multimodal vestibular cortex network (MVC), and brainstem–cerebellar network (BSC).

Group‐level ICA identified six ICNs across all participants: the DMN, VIS network, CB network, oculomotor/FEF network, MVC network, and a BSC network (Figure 2). The BSC component included pontomedullary brainstem regions together with CB midline structures, consistent with a ponto‐vermian/vestibuloCB circuit. Of the 25 components produced by GIFT/Infomax, brainstem‐related ICs were inspected blind by two neuro‐otologists (Sun‐Young Oh and Jin‐Ju Kang); the component showing an anatomically plausible BSC pattern and a dominant low‐frequency spectrum (0.01–0.05 Hz) was retained, whereas a pontine reticular/noise component was discarded.

**Figure 3:**
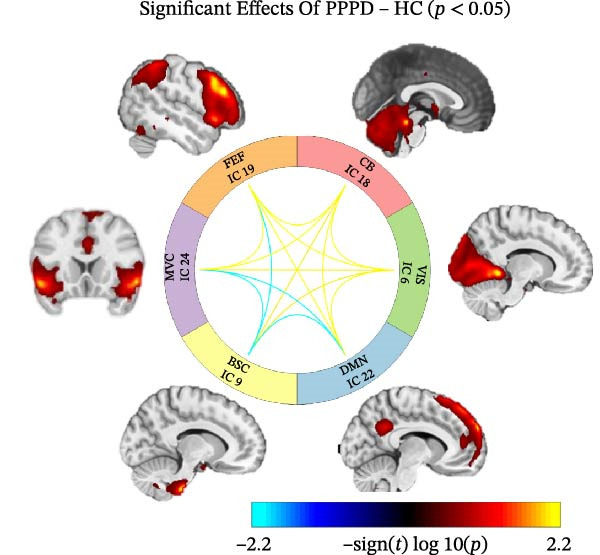
Functional network connectivity (FNC) alterations in patients with persistent postural‐perceptual dizziness (PPPD) relative to healthy controls (HCs). The analysis reveals statistically significant pairwise connectivity changes among key independent components, including the default mode (DMN), visual (VIS), brainstem–cerebellar (BSC), cerebellar (CB), frontal eye field (FEF), and multimodal vestibular cortex (MVC). A distinct double dissociation pattern is observed in PPPD patients. Increased connectivity (indicated by yellow or red lines) prominently involves the cerebellar (CB) and visual (VIS) networks, which show stronger coupling with all other networks (pFDR < 0.02), including a significant increase in BSC–VIS coupling (pFDR = 0.014). Conversely, decreased connectivity (indicated by blue lines) is identified between the BSC network and the multimodal vestibular cortex (MVC), frontal eye field (FEF), and default‐mode (DMN) networks (pFDR < 0.01). The thickness of each edge represents the corresponding effect size, and all displayed connections survive false discovery rate (FDR) correction (*q* < 0.05).

To examine internetwork dynamics, FNC matrices were generated and compared between PPPD patients and controls (Figure 3). Compared with HC, PPPD patients showed reduced connectivity between the BSC network and the MVC, FEF, and DMN networks (all pFDR < 0.01). In contrast, PPPD patients showed increased connectivity involving the CB network (with all other networks, all pFDR < 0.02) and the VIS network (with all other networks, all pFDR < 0.02), including increased BSC–VIS connectivity (pFDR = 0.014). These contrasts were unchanged after adjustment for age, sex, and head‐motion covariates.

Edge‐wise analyses (Figure 4) demonstrated that increased BSC–VIS connectivity was the only network metric that correlated with clinical measures within the PPPD group. PPPD patients showed stronger BSC–VIS connectivity than HC (*t* = 3.42, *p* = 0.001, Cohen’s *d* = 0.68). Within the PPPD group, higher BSC–VIS coupling correlated with greater depressive symptom severity (PHQ‐9: *r* = 0.48, pFDR = 0.012), higher state anxiety (STAI‐X1: *r* = 0.52, pFDR = 0.008), lower resilience (BRS: *r* = −0.45, pFDR = 0.018), and lower balance confidence (ABC: *r* = −0.50, pFDR = 0.010; Figure 4). All correlations remained significant after controlling for age, sex, and mean FD. No other FNC edge showed a clinical association that survived correction.

Figure 4Brain‐behavior coupling: BSC–VIS connectivity and clinical phenotype. Scatter plots showing the relationship between brainstem–cerebellar to visual network (BSC–VIS) functional connectivity strength and clinical measures within the PPPD group. Higher BSC–VIS coupling correlated with (A) greater depressive symptom severity (PHQ‐9: *r* = 0.48, pFDR = 0.012), (B) higher state anxiety (STAI‐X1: *r* = 0.52, pFDR = 0.008), (C) lower resilience (BRS: *r* = −0.45, pFDR = 0.018), and (D) lower balance confidence (ABC: *r* = −0.50, pFDR = 0.010). All correlations are partial Spearman correlations controlling for age, sex, and mean framewise displacement. *p*‐Values are FDR‐corrected for four planned comparisons (Benjamini–Hochberg, *q* < 0.05). BSC–VIS connectivity was significantly elevated in PPPD patients compared to healthy controls (inset: *t* = 3.42, *p* = 0.001).

**Figure:**
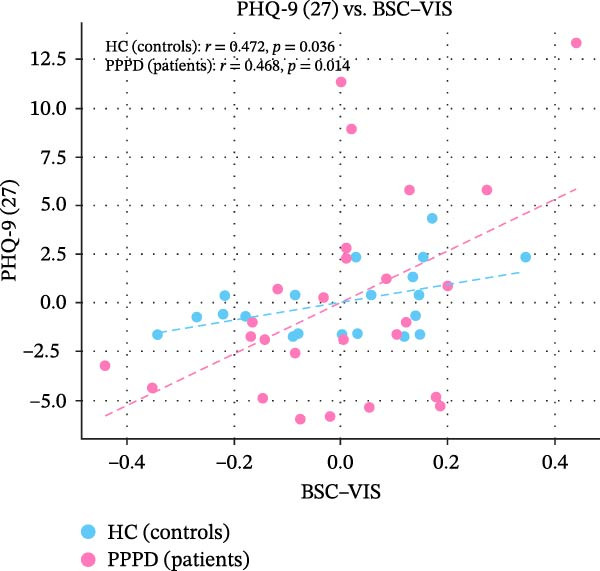
(A)

**Figure:**
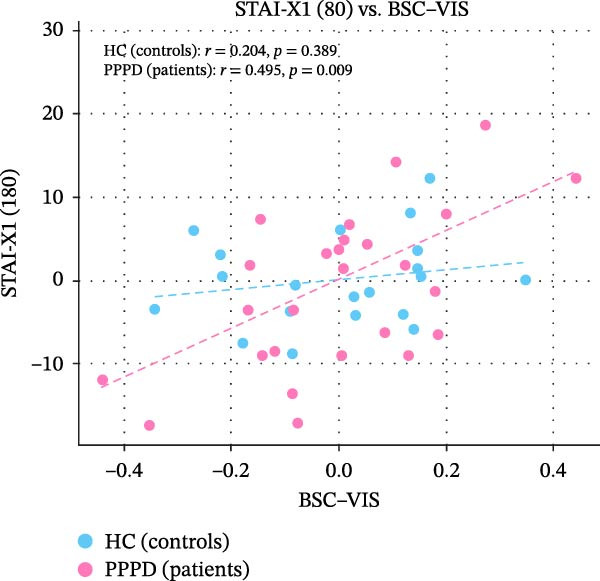
(B)

**Figure:**
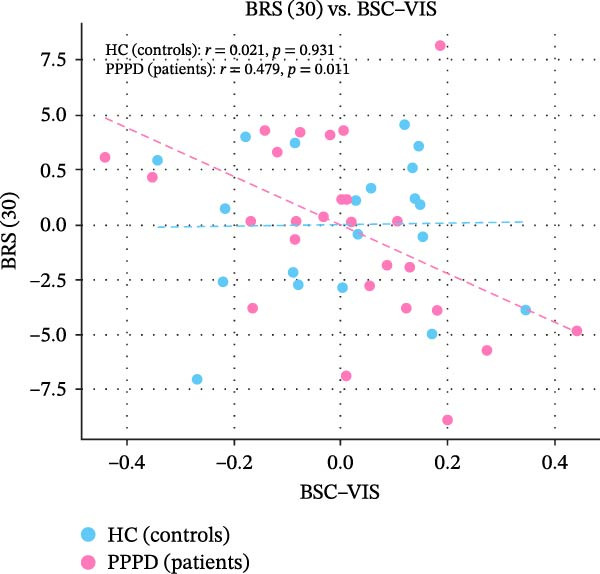
(C)

**Figure:**
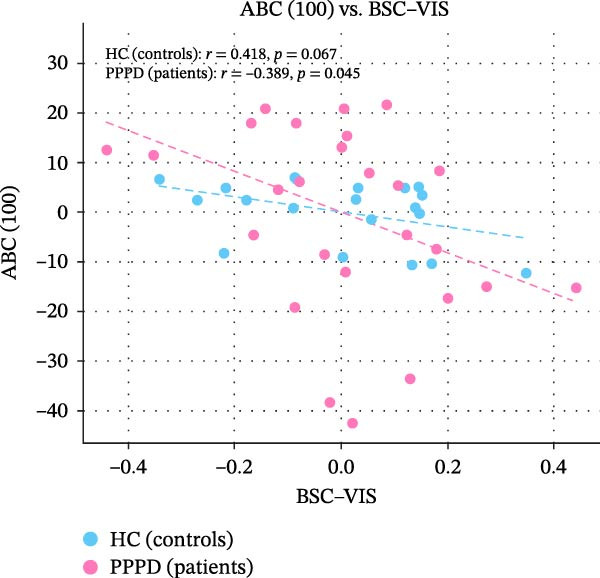
(D)

Voxel‐wise ALFF and fALFF analyses were conducted to assess regional spontaneous brain activity (Figure 5). Compared with HC, PPPD patients showed reduced ALFF in two clusters that form part of the MVC: the left parietal operculum (4649 voxels; peak MNI −39, −33, 22) and the right inferior frontal operculum/insula (425 voxels; peak 15, 27, 3); both survived whole‐brain family‐wise‐error correction at *p*  < 0.05. In contrast, PPPD patients showed elevated fALFF in three regions: mid‐cingulate gyrus / dorsal anterior cingulate (248 voxels; peak 0, 9, 36), left lateral occipital cortex (275 voxels; peak −51, −75, 15), and right precentral gyrus (190 voxels; peak 45, −12, 42), cluster‐corrected at *p*  < 0.05. Age, sex, and mean FD were included as covariates in all analyses. No other brain regions showed significant differences between groups at the specified threshold.

Figure 5Group differences in intrinsic oscillatory amplitude. Whole‐brain voxel‐wise contrasts of resting‐state amplitude of low‐frequency fluctuations (ALFF) and fractional ALFF (fALFF) are shown on the Montreal Neurological Institute (MNI‐152) template; statistical maps are cluster‐level family‐wise‐error corrected at *p*  < 0.05 (height threshold *p* < 0.001, two‐tailed). Warmer colors denote a larger between‐group difference; slice coordinates are in MNI space and numbers on the color bar index clusters listed below. (A) ALFF: healthy controls > PPPD. Two significant clusters indicated lower spontaneous power in patients: (A1) Left parietal operculum / posterior insula / secondary somatosensory cortex (4649 voxels; peak −39, −33, 22). (A2) Right inferior frontal operculum (425 voxels; peak 15, 27, 3). (B) fALFF: PPPD > healthy controls. Three clusters showed relatively higher fractional power in patients: (B1) Mid‐cingulate gyrus (248 voxels; peak 0, 9, 36)—region linked to interoception and affective regulation. B2. Left lateral occipital cortex (275 voxels; peak −51, −75, 15)—involved in visual–spatial processing. (B3) Right precentral gyrus (primary motor cortex) (190 voxels; peak 45, −12, 42)—consistent with motor vigilance. Collectively, the pattern depicts diminished activity in cortical vestibular integration hubs alongside heightened visual, motor and interoceptive drive in persistent postural‐perceptual dizziness. ALFF, amplitude of low‐frequency fluctuations; fALFF, fractional ALFF; PPPD, persistent postural‐perceptual dizziness; HC, healthy control.

**Figure:**
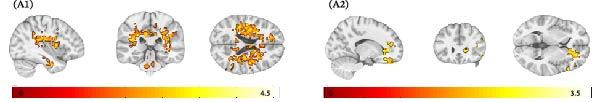
(A)

**Figure:**
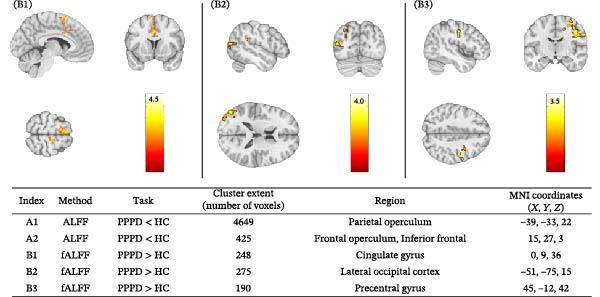
(B)

**Figure 6:**
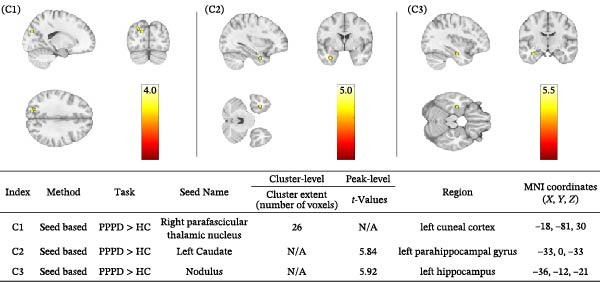
Seed‐based functional connectivity analysis identifying regions where patients with persistent postural‐perceptual dizziness (PPPD) exhibited significantly higher functional connectivity compared to healthy controls (HC). Three seed regions showed significant findings (*p*  < 0.05, FWE‐corrected). C1: Right parafascicular thalamic nucleus showed cluster‐level significance with the left cuneal cortex (26 voxels; MNI: −18, −81, 30). C2: Left caudate nucleus exhibited peak‐level significance with the left parahippocampal gyrus (*t* = 5.84; MNI: −33, 0, −33). C3: Cerebellar nodulus demonstrated peak‐level significance with the left hippocampus (*t* = 5.92; MNI: −36, −12, −21). These findings suggest altered connectivity within vestibulo‐limbic and spatial memory circuits in PPPD. Accompanying brain images show the significant connectivity patterns in axial, coronal, and sagittal planes. Warmer colors represent greater differences in connectivity strength. Numbers on the brain images correspond to the seed regions listed in the table. ‘N/A’ indicates no significant cluster or peak result for a given level of analysis.

Seed‐based connectivity analysis was performed to examine circuit‐level alterations (Figure 6). Relative to controls, PPPD patients exhibited significantly increased functional connectivity between (C1) the right parafascicular thalamus and left cuneal cortex (MNI: −18, −81, 30; cluster‐level pFWE = 0.020), (C2) the left caudate and left parahippocampal gyrus (MNI: −33, 0, −33; peak‐level pFWE = 0.019), and (C3) the CB nodulus and left hippocampus (MNI: −36, −12, −21; peak‐level pFWE = 0.015). All analyses included age, sex, and mean FD as covariates.

## 5. Discussion

Building on prior resting‐state fMRI evidence that visuo‐vestibular and limbic networks are implicated in PPPD [12, 55, 59, 74–77], we applied a multilevel resting‐state framework in a well‐characterized cohort (52 PPPD; 50 HCs). Across complementary network‐level (ICA‐based FNC), regional (ALFF/fALFF), and circuit‐level (seed‐based connectivity) analyses, we observed a convergent configuration consistent with maladaptive sensory reweighting. At the network level, PPPD was characterized by reduced coupling of the BSC network with the MVC, oculomotor/FEF, and DMN, alongside broadly increased CB and VIS network connectivity (Figure 3). Regional amplitude mapping showed reduced ALFF in opercular vestibular hubs with increased fALFF in cingulate, lateral occipital, and precentral cortices (Figure 5). Circuit‐level analyses further demonstrated strengthened thalamo‐VIS, striato‐limbic, and nodulus–hippocampal connectivity (Figure 6). Importantly, increased BSC–VIS coupling was the only ICA‐FNC metric that tracked clinical phenotype, correlating with depressive and anxiety symptoms, lower resilience, and reduced balance confidence (Figure 4).

Prior rs‐fMRI studies of PPPD have generally examined small samples (typically 11–38 cases) using single‐tier analytic pipelines, producing heterogeneous—and at times apparently opposing—findings (Table 4). Lee et al. [12] and Li et al. [19] reported reduced connectivity or amplitude in opercular/precuneus regions implicated in multisensory integration, whereas Li et al. (ICA) [20], Yagi et al. [18], and Liu et al. [78] emphasized increased VIS‐CB coupling and/or precuneus hyperactivity. In our cohort, we observed vestibular cortical downregulation alongside VIS/CB overconnectivity, suggesting that these patterns can co‐occur within the same dataset rather than being mutually exclusive. By integrating ICA‐FNC, ALFF/fALFF and seed‐based analyses, we show that opercular hypoactivity can coexist with network‐level VIS‐CB hyperconnectivity and limbic‐linked circuit reinforcement, offering a parsimonious framework that can reconcile prior variability across methods and cohorts.

**Table 4:** Published resting‑state fMRI studies in PPPD: cohorts, analytic tiers, and principal findings.

Year	First author/journal	Cohort (PPPD/HC)	Main analytic tier	Key findings
2018	Lee et al. [12] Human Brain Mapping	38/38	Seed‑ & ROI‑based FC	↓ Hippocampus ↔ opercular‑insula and vestibular hubs; ↑ sub‑callosal ↔ visual‑frontal coupling; FC alterations predicted symptom severity
2020	Li et al. [20] NeuroImage: Clinical	20/20	ICA‑FNC	↓ Default‑mode precuneus coherence; ↑ occipital‑pole visual links with auditory / sensorimotor networks; authors interpreted as visual substitution
2020	Li et al. [19] Brain Imaging Behav	16/18	ALFF / ReHo	Hypo‑ALFF in right precuneus + cuneus; magnitude correlated with DHI score, supporting vestibular cortical under‑activity hypothesis
2023	Yagi et al. [18] Frontiers Neurol	15/18	ICA before & after optokinetic stimulus	Baseline hyper‑connectivity among vestibulo‑visual‑somatosensory nodes that further increased after visual motion, highlighting visual dependence
2024	Liu et al. [78] Brain Connectivity	30/30	fALFF + VMHC	↑ fALFF/↓ homotopy in bilateral precuneus; fALFF positively related to DHI, while homotopic loss tracked disease duration

Abbreviations: ALFF, amplitude of low‑frequency fluctuations; ALFF, fractional ALFF; DHI, dizziness handicap inventoryf; FC, functional connectivity; FNC, functional network connectivity; HC, healthy control; ICA, independent‑component analysis; PPPD, persistent postural‑perceptual dizziness; ReHo, regional homogeneity; ROI, region of interest; VMHC, voxel‐mirrored homotopic connectivity.

From a vestibular systems perspective, brainstem vestibular pathways and their CB projections constitute an early relay for multisensory balance information [79, 80]. The reduced BSC coupling with cortical systems supporting vestibular integration (MVC), eye‐movement control (FEF), and self‐referential processing (DMN) is compatible with diminished propagation or utilization of vestibular signaling at the network level in PPPD [8, 11]. In response, the system may up‐weight CB predictive models and VIS motion input, reflected in CB/VIS hyperconnectivity and strengthening of the BSC–VIS edge [81, 82]. This interpretation aligns with behavioral reports of increased VIS dependence and symptom exacerbation in visually complex environments [55, 83]. Together, the network‐level double dissociation—reduced BSC coupling to vestibular/oculomotor/DMN systems but increased coupling involving VIS and CB networks—supports a maladaptive rebalancing of sensory weights rather than simple loss of vestibular function.

Regional amplitude mapping refined this network‐level account. Suppressed ALFF in parietal and frontal opercular cortices—core hubs of the human vestibular cortex—suggests reduced intrinsic readiness to integrate vestibular inputs. Conversely, elevated fALFF in dorsal anterior cingulate (salience/monitoring), lateral occipital (VIS), and precentral (premotor) cortices may reflect heightened top–down monitoring and preparatory motor control in response to perceived postural threat [84, 85]. Seed‐based connectivity further extended the framework to subcortical loops. Increased coupling of the parafascicular thalamus with the VIS cortex, the caudate with the parahippocampal cortex, and the nodulus with the hippocampus links vestibular–CB processing to circuits supporting sensory gating, action selection, contextual memory, and affective salience. Such reinforcement may provide a pathway through which ambiguous motion cues acquire anxiety‐laden valence and perpetuate anticipatory postural tension—consistent with patients’ descriptions of “constant readiness to fall.” [74, 86] Notably, Our multitiered findings converge on a common pathophysiological theme: a breakdown in early‐stage sensory filtering that drives higher‐order maladaptation. While prior research has emphasized cortical hubs like the PIVC, our results highlight that functional alterations in the BSC–VIS network and specific subcortical circuits (e.g., parafascicular thalamus and caudate) may serve as the primary ‘bottom‐up’ drivers of the disorder. Specifically, the strengthened coupling between subcortical gatekeepers and VIS/limbic regions suggests that vestibular and VIS signals are inappropriately filtered before reaching cortical awareness. This subcortical dysregulation likely fuels the maladaptive cortical reweighting and top–down oculomotor instability (FEF) characteristic of PPPD. By identifying these disturbances at the brainstem and thalamo‐striatal levels, we provide a more comprehensive framework for how distorted sensory signals are amplified into persistent unsteadiness and VIS vertigo.

Psychometric profiling in our cohort confirmed the classic clinic–laboratory mismatch: PPPD patients reported marked handicap and reduced balance confidence despite largely normal vestibular tests. These behavioral findings are consistent with predictive‐coding accounts in which the brain over‐weights internally generated predictions while discounting veridical vestibular input, producing a persistent error signal consciously experienced as unsteadiness [87, 88]. We also observed meaningful links between affective traits and symptom appraisal: depressive symptoms related to higher dizziness handicap, neuroticism related to lower balance confidence, and resilience showed a protective association with balance confidence and functional handicaps. These patterns align with biopsychosocial models that position emotional vulnerability and maladaptive coping as key drivers of symptom persistence [74, 89]. Crucially, our brain‐behavior analyses connected these domains: stronger BSC–VIS coupling correlated with higher depression and state anxiety, lower resilience, and lower balance confidence. This suggests a plausible neural route by which affective distress may bias sensory weighting toward VIS processing, reinforcing the vicious cycle proposed in PPPD (VIS dependence, hypervigilance, and anxiety mutually amplifying symptoms) [2]. While these associations do not establish causality, they motivate future work testing whether interventions that reduce affective distress or VIS dependence also normalize BSC–VIS coupling.

Clinically, these findings support conceptualizing PPPD as a distributed network imbalance rather than a focal vestibular deficit. If replicated, multiscale network measures—particularly BSC–VIS coupling—could contribute to objective stratification (e.g., identifying patients with prominent affect‐modulated VIS dependence) and to mechanistic monitoring of treatment response. However, these measures are not yet suitable for individual‐level diagnosis, and their value will depend on external validation, assessment of test–retest reliability, and demonstration of sensitivity to clinically meaningful change.

Using *a* ~6 min resting‐state scan, we derived connectivity and amplitude patterns that differentiated PPPD from controls, but the present results remain exploratory and require replication in larger, multicenter datasets. Future longitudinal and interventional studies should test whether established treatments (e.g., vestibular rehabilitation, graded VIS desensitization, and psychological therapies) normalize the network markers identified here and whether marker change tracks symptom trajectories. Task‐based paradigms that probe VIS motion processing, postural threat, or vestibular stimulation may further clarify how these resting‐state alterations translate into symptom provocation and avoidance behavior.

Several limitations should be acknowledged. First, the cross‐sectional design precludes causal inference; longitudinal imaging is needed to determine whether BSC–VIS hypercoupling precedes chronicity or reflects secondary adaptation. Second, we did not acquire field maps for susceptibility distortion correction, and conventional rs‐fMRI resolution limits precise localisation within the brainstem; accordingly, the BSC component should be interpreted at the network level rather than as an isolated vestibular nuclei signal. Third, although stringent motion control was applied, residual physiological noise may disproportionately affect brainstem signals. Fourth, single‐center recruitment and exclusion of severe psychiatric comorbidity may limit generalisability. Fifth, we cannot fully disentangle the influence of migraine or psychotropic medication exposure, and affective state was captured by questionnaires rather than ecological sampling. Furthermore, while our ICA‐FNC analysis prioritized six networks (DMN, VIS, CB, FEF, MVC, and BSC) based on their core roles in the visuo‐vestibular axis, we acknowledge that others, such as the sensorimotor and limbic networks, were not included in the final FNC model. Although these networks are implicated in PPPD, they were not included in this targeted ICA‐FNC tier to reduce the multiple‐comparison burden and to maintain a focused test of the visuo‐vestibular reweighting hypothesis; future work should evaluate broader FNC models that explicitly incorporate sensorimotor and limbic networks. Finally, the eyes‐closed resting‐state protocol may limit generalizability to eyes‐open conditions that are more directly related to visually provoked symptoms. In addition, the 3T MRI environment itself introduces a potential confounding factor known as magnetic vestibular stimulation (MVS). As highlighted by recent studies, static magnetic fields can induce subclinical nystagmus and modulate fluctuations in oculomotor and DMNs, even with eyes closed [90, 91]. While this effect was present for all participants, our findings of significant group differences in FEF connectivity may reflect a disease‐specific neural susceptibility or maladaptive response to these constant, low‐level vestibular inputs in PPPD patients.

In summary, PPPD was associated with reduced coupling between a BSC network and multimodal vestibular/oculomotor systems, concurrent CB‐VIS overintegration, and strengthened limbic‐associated circuits. The association of BSC–VIS coupling with depression, anxiety, resilience, and balance confidence supports a mechanistic link between affective traits and maladaptive sensory weighting. Replication and longitudinal validation are needed to establish the clinical utility of these network‐level markers and to inform network‐targeted therapeutic strategies.

## Acknowledgments

This work was supported by the National Research Foundation of Korea (NRF) grant funded by the Korean Government (Ministry of Science and ICT) (Grant RS‐2025‐00553480) and by a grant of the Korea Health Technology R&D Project through the Korea Health Industry Development Institute (KHIDI), funded by the Ministry of Health & Welfare, Republic of Korea (Grant RS‐2025‐02223226). This research was also supported by the Biomedical Research Institute Fund, Jeonbuk National University Hospital.

## Funding

This study was funded by the Ministry of Science and ICT, South Korea (Grant RS‐2025‐00553480), the Korea Health Industry Development Institute (Grant RS‐2025‐02223226), and the Biomedical Research Institute of Jeonbuk National University Hospital.

## Conflicts of Interest

The authors declare no conflicts of interest.

## Supporting Information

Additional supporting information can be found online in the Supporting Information section.

## Supporting information


**Supporting Information 1** Figure S1: Spatial Distribution of All 25 ICA Components. Individual panels show the mean spatial maps for each of the 25 ICs obtained from group ICA. To ensure full data transparency, all identified components—including those not included in the primary ICA‐FNC model—are presented. Six intrinsic connectivity networks (ICNs) prioritized for the targeted functional network connectivity (FNC) analysis are distinguished by colored title bars: VIS (IC 6), BSC (IC 9), CB (IC 18), FEF (IC 19), DMN (IC 22), and MVC (IC 24). The remaining components include additional canonical brain networks and artifact‐related components (e.g., edge/motion, CSF/vascular), which were not included in this focused FNC model (see Methods for exclusion criteria).

**Supporting Information 2** Table S1. Clinical features of PPPD patients. Table S2. ROI definitions and atlas sources for seed‐based functional connectivity analyses.

## Data Availability

Anonymised data underlying the findings of this study, including imaging results, clinical measures, and statistical outputs, are available from the corresponding author upon reasonable request. Data sharing is subject to institutional and ethical approvals in accordance with participant consent and local data protection regulations.
