# Supplementary figures and images for: Vestibular‐Visual Reweighting in Persistent Postural‐Perceptual Dizziness: A Multilevel Resting‐State fMRI Study
Source: Neural Plast. 2026 Apr 8;2026:9968808. doi: 10.1155/np/9968808 (PMC13058442; doi:10.1155/np/9968808)

## Slide 1
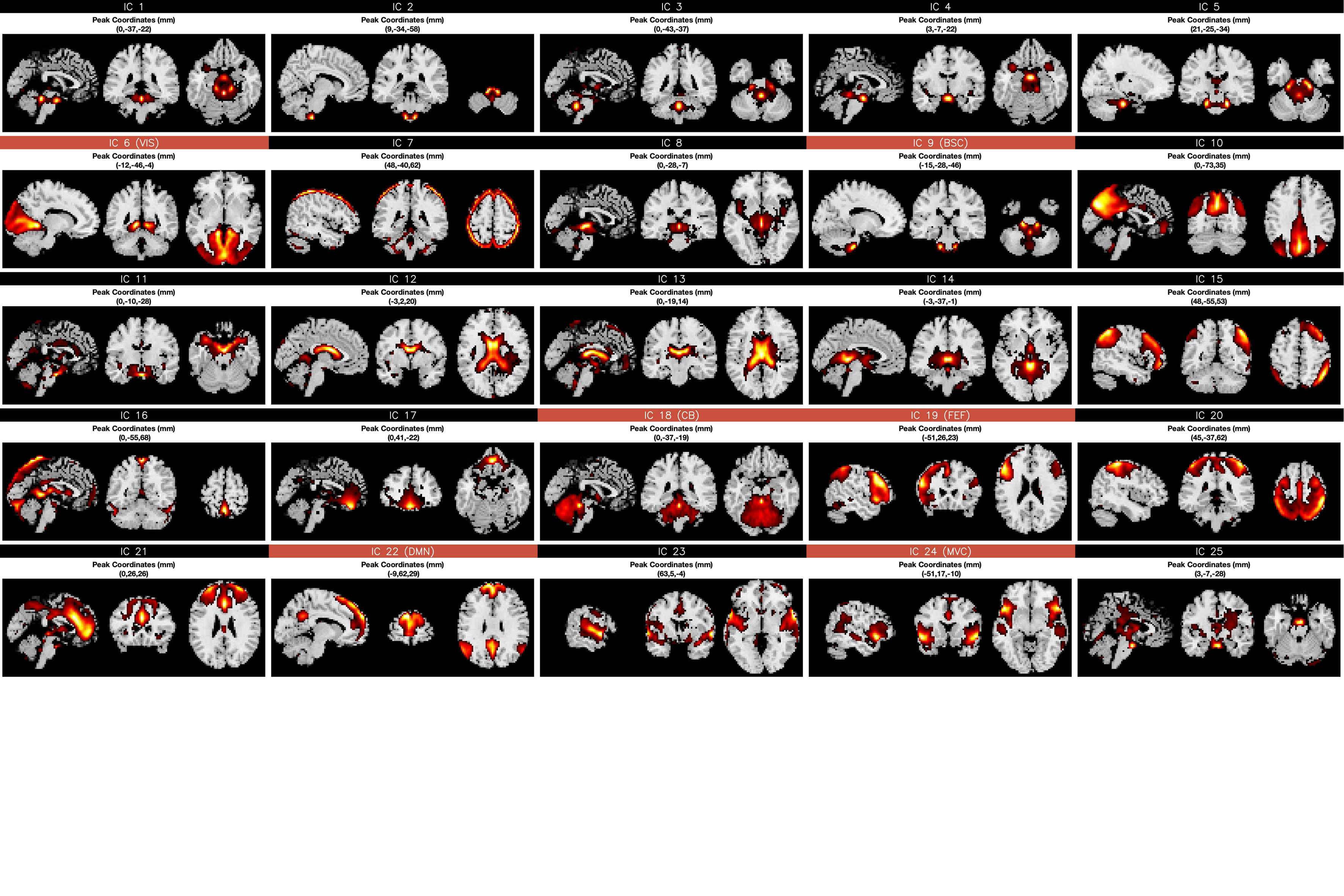

Supplement: Supplementary file 1 — Supporting Information 1 Figure S1: Spatial Distribution of All 25 ICA Components. Individual panels show the mean spatial maps for each of the 25 ICs obtained from group ICA. To ensure full data transparency, all identified components—including those not included in the primary ICA‐FNC model—are presented. Six intrinsic connectivity networks (ICNs) prioritized for the targeted functional network connectivity (FNC) analysis are distinguished by colored title bars: VIS (IC 6), BSC (IC 9), CB (IC 18), FEF (IC 19), DMN (IC 22), and MVC (IC 24). The remaining components include additional canonical brain networks and artifact‐related components (e.g., edge/motion, CSF/vascular), which were not included in this focused FNC model (see Methods for exclusion criteria). [file NP-2026-9968808-s001.pptx]
