# Supplementary material for: Vestibular‐Visual Reweighting in Persistent Postural‐Perceptual Dizziness: A Multilevel Resting‐State fMRI Study
Source: Neural Plast. 2026 Apr 8;2026:9968808. doi: 10.1155/np/9968808 (PMC13058442; doi:10.1155/np/9968808)
Supplement: Supplementary file 2 — Supporting Information 2 Table S1. Clinical features of PPPD patients. Table S2. ROI definitions and atlas sources for seed‐based functional connectivity analyses. [file NP-2026-9968808-s002.docx]

Supplementary Table 1. Clinical features of PPPD patients

|  | Core vestibular symptoms | | Getting worse  over time | Precipitating factors | Aggravating factors | | | Relieving factors | Vestibular function tests | | |
| --- | --- | --- | --- | --- | --- | --- | --- | --- | --- | --- | --- |
|  | Unsteadiness | Dizziness/  Vertigo |  |  | Upright posture | Active or passive motion | Complex visual stimuli |  | Caloric paresis, % | vHIT HC gain, R/L | oVEMP  /cVEMP |
| HC  (n=50) | - | - | - | - | - | - | - | - | 6.61±8.9 | 0.97±0.05/0.98±0.06 | Normal  /Normal |
| P1 | Wobbling, Unsteadiness | Spinning type dizziness | Yes | Panic disorder, Emotional stress | Sitting up, standing  without support | Head moving | Using a computer or smartphone | Lying | 17.16 | 0.95/0.96 | Normal  /Normal |
| P2 | Feeling of fall | Spinning type dizziness | Yes | Panic disorder | Sitting up | Riding in a transportation | Seeing a lot of stairs | Lying | -6.98 | 1.01/1.06 | Normal  /Normal |
| P3 | Feeling of fall | Non-spinning type dizziness | - | BPPV | Sitting up | Head moving | Watching clouds or moving swing | Lying | 6.20 | 1.02/0.97 | NR, L  /Normal |
| P4 | Feeling of veering | Spinning type dizziness | Yes | Trauma | Sitting up | Body moving | Seeing a zigzag pattern | Lying, sitting | -12.24 | 0.95/0.94 | Normal  /Normal |
| P5 | Floating | Spinning type dizziness | - | BPPV, Emotional stress | Sitting up | Head moving | Reading small letters | Lying, talking | -20.00 | 1.02/0.96 | Normal  /Normal |
| P6 | Wobbling | Non-spinning type dizziness, feeling faint | Yes | Syncope, Anemia | Sitting up, standing  without support | Riding in a transportation,  looking at a moving object | Using a computer or smartphone | Sitting | -15.68 | 0.98/1.06 | Normal  /Normal |
| P7 | Wobbling | Non-spinning type dizziness | Yes | Emotional stress | Sitting up | Head moving | Using a computer or smartphone,  reading small letters | Talking | -7.05 | 0.95/0.89 | Normal  /Normal |
| P8 | Drunken | Non-spinning type dizziness | - | VN, Migraine, Emotional stress | Sitting up, walking | Head moving, body moving | Using a computer or smartphone | Lying, sitting, talking | -29.22 | 0.41/0.70 | Delayed, L  /Delayed, R |
| P9 | Feeling of fall | Non-spinning type dizziness, cloudiness | - | BPPV | Sitting up | Body moving | Seeing crowded people, movie screen | Sitting | 2.75 | 0.91/0.91 | Delayed, L  /Normal |
| P10 | Feeling of fall | Spinning type dizziness | - | BPPV | Walking | Body moving | Using a computer or smartphone | Talking | 18.35 | 1.02/0.90 | Normal  /Normal |
| P11 | Drunken | Spinning type dizziness, nausea | - | BPPV, Panic disorder | When walking | Head moving,  Riding in a transportation | Using a computer or smartphone | Lying, talking | 13.36 | 0.97/0.99 | Normal  /Normal |
| P12 | Wobbling | Non-spinning type dizziness | - | VN, Panic disorder | Standing | Body moving | Using a computer or smartphone | Lying, sitting | 27.47 | 0.88/0.66 | Normal  /Normal |
| P13 | Wobbling | Spinning type dizziness | - | BPPV, Emotional stress | Sitting up | Going up the stairs,  crossing the bridge | Watching the television | Lying | 9.04 | 1.09/0.99 | Normal  /Normal |
| P14 | Drunken, Unsteadiness | Non-spinning type dizziness | - | Panic disorder | Sitting up | Head moving, body moving,  riding in a transportation | Using a computer or smartphone | Lying | 10.61 | 1.01/0.95 | -/- |
| P15 | Wobbling | Spinning type dizziness | - | BPPV, MD | Sitting up, walking | Head moving, body moving,  riding in a transportation | Using a computer or smartphone | Lying, sitting | - | 0.89/0.88 | -/- |
| P16 | Feeling of fall | Non-spinning type dizziness, feeling faint | Yes | VN, Visual stimuli | Sitting up, walking | Riding in a transportation | Using a computer or smartphone,  reading small letters | Sitting | 42.88 | 0.92/0.36 | Delayed, L  /Normal |
| P17 | Wobbling | Non-spinning type dizziness | - | VN | Standing without support | Body moving,  riding in a transportation | Using a computer or smartphone | Lying | 15.15 | 0.97/1.00 | Normal  /Normal |
| P18 |  | Spinning type dizziness | - | BPPV | Sitting up | Head moving | Seeing crowded people | Sitting | 24.41 | 0.93/0.87 | Normal  /Normal |
| P19 | Wobbling | Spinning type dizziness | - | - | Standing without support, walking | Head moving | Reading small letters |  | - | 0.99/0.97 | -/- |
| P20 | Wobbling | Non-spinning type dizziness | Yes | - | Sitting up, standing  without support | Head moving,  riding in a transportation | Watching the television, watching a rapidly rotating object | Lying, talking | -4.46 | 1.01/0.94 | Normal  /Delayed, R |
| P21 | Wobbling  Feeling of fall | Non-spinning type dizziness | - | Emotional stress | Sitting up | Head moving | Using a computer or smartphone, watching the television | Sitting | - | 0.95/0.96 | Normal  /Normal |
| P22 | Feeling of fall | Non-spinning type dizziness | Yes | Panic disorder, Emotional stress | Standing without support | Head moving | Seeing a repeating pattern, using a computer or smartphone | Lying | - | 0.81/0.94 | Normal  /Normal |
| P23 | Wobbling | Non-spinning type dizziness | - | Emotional stress | Sitting up, standing  without support, walking | Head moving,  riding in a transportation | Using a computer or smartphone, reading small letters | Lying | - | 0.90/0.96 | -/- |
| P24 | Wobbling | Non-spinning type dizziness | - | Emotional stress | Sitting up, standing  without support | Head moving | Using a computer or smartphone | Lying | - | 0.99/0.89 | -/- |
| P25 | Feeling of fall | Non-spinning type dizziness | Yes | BPPV, Migraine | Sitting up, standing  without support, walking | Head moving,  riding in a transportation | Using a computer or smartphone, reading small letters | Lying, Sitting | 21.64 | 0.91/0.83 | Delayed, L  /Delayed, R |
| P26 | Wobbling | Spinning type dizziness | Yes | BPPV, Emotional stress | Standing without support | Head moving | Seeing a repeating pattern | Lying, Sitting | 7.60 | 1.09/1.13 | Normal  /Normal |
| P27 | Wobbling, Unsteadiness | Spinning type dizziness | - | BPPV | Concentration, walking | Head moving, body moving | Using a computer or smartphone | Sitting, not move | - | 0.99/0.97 | -/- |
| P28 | Feeling of fall | Non-spinning type dizziness, feeling faint | - | - | Standing, walking | Body moving | Using a computer or smartphone,  reading small letters | Lying | - | 1.01/0.94 | -/- |
| P29 | Feeling of fall | Non-spinning type dizziness | - | - | Standing, walking | Body moving | Using a computer or smartphone | Lying | - | 0.95/0.96 | Normal  /Normal |
| P30 | Feeling of veering | Non-spinning type dizziness | Yes | Emotional stress | Standing | Head moving,  Riding in a transportation | Seeing crowded people | Lying | 29.4 | 0.81/0.94 | -/- |
| P31 | Floating | Non-spinning type dizziness, cloudiness | - | Panic disorder, Emotional stress | Sitting up | Head moving, body moving | Reading small letters, using smart phone | Lying, Sitting | 5.61 | 0.99/0.97 | -/- |
| P32 | Wobbling | Spinning type dizziness | - | Emotional stress | Sitting up | Head moving, body moving | Using a computer or smartphone | Sitting | - | 0.89/0.98 | Delayed, L  /Delayed, R |
| P33 | Wobbling | Non-spinning type dizziness, feeling faint | - | Emotional stress | Sitting up, walking | Body moving | Using a computer or smartphone | Sitting, not move | - | 0.90/1.00 | -/- |
| P34 | Drunken, Unsteadiness | Non-spinning type dizziness | Yes | BPPV, Migraine | Sitting up, walking | Head moving,  Riding in a transportation | Using a computer or smartphone,  reading small letters | Lying | - | 0.95/1.00 | Normal  /Normal |
| P35 | Feeling of fall | Non-spinning type dizziness | - | Emotional stress | Standing | Body moving | Using a computer or smartphone | Lying | 30.0 | 0.92/0.89 | Normal  /Normal |
| P36 | Feeling of fall | Spinning type dizziness | Yes | - | Concentration, walking | Head moving, body moving | Escalator, Seeing crowded people | Lying | 17.7 | 0.99/0.99 | -/- |
| P37 | Drunken, Feeling of fall | Non-spinning type dizziness | - | Emotional stress | Sitting up, walking | Head moving | Reading small letters, using smart phone | Sitting, not move | 10.61 | 0.95/0.90 | Normal  /Normal |
| P38 | Drunken, Feeling of fall | Non-spinning type dizziness | - | Emotional stress | Sitting up, walking | Head moving | Reading small letters, using smart phone | Sitting, not move | 12.1 | 0.95/0.90 | Normal  /Normal |
| P39 | Wobbling | Non-spinning type dizziness | - | BPPV | Sitting up, standing  without support walking | Head moving, body moving | Using a computer or smartphone, watching the television | Lying | 30.0 | 0.95/0.90 | Normal  /Normal |
| P40 | Wobbling | Spinning type dizziness | - | - | Sitting up, standing  without support | Head moving | Using a computer or smartphone, watching the television | Lying | 15.05 | 0.87/0.89 | -/- |
| P41 | Wobbling | Non-spinning type dizziness | Yes | BPPV | Sitting up | Head moving, body moving | Reading small letters, using smart phone | Lying | 20.62 | 0.99/0.98 | Normal  /Normal |
| P42 | Wobbling | Non-spinning type dizziness | - | MD | Sitting up | Head moving, body moving | Reading small letters, using smart phone | Lying | - | 0.95/0.44 | -/- |
| P43 | Feeling of veering | Non-spinning type dizziness | - | VN | Sitting up, standing  without support | Head moving, body moving | Seeing a fall, seeing in a height | Lying, Sitting |  | 0.39/0.98 | -/- |
| P44 | Feeling of fall | Spinning type dizziness | - | Emotional stress | Sitting up, walking | Head moving, body moving | Seeing in a height | Lying, Sitting | 10.6 | 0.91/0.89 | Delayed, L  /Delayed, R |
| P45 | Feeling of fall | Cloudiness | Yes | Emotional stress, bad weather | Sitting up | Body moving | Using a computer or smartphone, watching the television | Lying | 9.0 | 0.85/0.90 | Normal  /Normal |
| P46 | Feeling of veering | Cloudiness | - | - | Sitting up | Head moving | Using a computer or smartphone, watching the television | Lying | 14.0 | 0.55/0.91 | Normal  /Normal |
| P47 | Feeling of fall | Non-spinning type dizziness, cloudiness | - | Emotional stress | Sitting up, standing  without support | Head moving, body moving | Using a computer or smartphone, watching the television | Lying | 21.11 | 0.95/0.90 | Normal  /Normal |
| P48 | Feeling of fall | Non-spinning type dizziness | - | VN, | Sitting up, walking | Body moving | Seeing a movie, Reading small letters | Lying, Sitting | 60.3 | 0.95/0.30 | Normal  /Normal |
| P49 | Feeling of fall | Non-spinning type dizziness | Yes | MD | Sitting up | Body moving | Seeing a fall | Lying, Sitting | 42.56 | 0.45/0.90 | NR, L  /Normal l |
| P50 | Feeling of fall | Spinning type dizziness | Yes | Bad weather | Sitting up, walking | Head moving, body moving | Seeing in a height | Sitting, not move | - | 0.96/0.92 | Normal  /Normal |
| P51 | Feeling of fall | Spinning type dizziness | Yes | Emotional stress, panic attack | Sitting up, standing  without support, walking | Head moving | Reading small letters, using smart phone | Sitting, not move | - | 0.89/0.90 | NR, R  /Normal |
| P52 | Feeling of veering | Spinning type dizziness, cloudiness | Yes | Emotional stress, BPPV | Sitting up, walking | Head moving | Using a computer or smartphone, watching the television | Sitting, not move | - | 0.99/0.98 | Reduced R  /Normal |

BPPV=Benign paroxysmal positional vertigo; VN=Vestibular neuritis; MD=Meniere’s disease; vHIT HC=video head impulse test at horizontal canal; oVEMP=ocular vestibular evoked myogenic potential; cVEMP=cervical vestibular evoked myogenic potential

Supplementary Table 2. ROI definitions and atlas sources for seed-based functional connectivity analyses

| Domain | ROI | Laterality | Atlas / template | Atlas label / definition | Ref. |
| --- | --- | --- | --- | --- | --- |
| Vestibular (cortical) | Parieto-insular vestibular cortex (PIVC; OP2) | Bilateral | Jülich histological atlas | OP2 parcel | 55 |
| Vestibular (cortical) | Inferior parietal lobule (IPL) | Bilateral | Harvard–Oxford Cortical Atlas | Inferior parietal lobule | 59 |
| Vestibular (cerebellar) | Nodulus (lobule X) | Midline | SUIT cerebellar atlas | Lobule X | 57 |
| Vestibular (cerebellar) | Flocculus | Bilateral | SUIT cerebellar atlas | Flocculus | 57 |
| Affective / limbic | Amygdala | Bilateral | AAL3 atlas | Amygdala | 58 |
| Affective / limbic | Hippocampus | Bilateral | AAL3 atlas | Hippocampus | 58 |
| Affective / limbic | Parahippocampal gyrus | Bilateral | AAL3 atlas | Parahippocampal gyrus | 58 |
| Affective / limbic | Anterior cingulate cortex (ACC) | Bilateral | Harvard–Oxford Cortical Atlas | Cingulate gyrus, anterior division | 59 |
| Affective / limbic | Medial prefrontal cortex (mPFC) | Bilateral | Harvard–Oxford Cortical Atlas | Frontal medial cortex | 59 |
| Affective / limbic | Insula | Bilateral | Harvard–Oxford Cortical Atlas | Insular cortex | 59 |
| Subcortical relay hub | Parafascicular thalamic nucleus | Bilateral | Stereotactic thalamic atlas (Morel/Krauth) | Parafascicular nucleus | 60 |
| Subcortical relay hub | Caudate nucleus | Bilateral | AAL3 atlas | Caudate | 58 |

Abbreviations: AAL3, Automated Anatomical Labeling atlas 3; SUIT, Spatially Unbiased Infratentorial Template; ROI, region of interest.
